# The individual patterns of anxieties and defense mechanisms in COVID pandemic context

**DOI:** 10.1192/j.eurpsy.2021.730

**Published:** 2021-08-13

**Authors:** E. Sokolova, A. Ryzhov, L. Pechnikova

**Affiliations:** Faculty Of Psychology, Lomonosov MSU, Moscow, Russian Federation

**Keywords:** Covid, mentalization, psychological defences, self-regulation

## Abstract

**Introduction:**

The COVID-pandemic context is characterized by a global ambiguity, reflecting the unexpected onset of pandemic, unpredictability and amplitude of the related dangers, questionability and the side effects of the policy measures used to protect people.

**Objectives:**

The development of theoretical framework for understanding variability in the reactions to COVID situation and self-regulatory mechanisms.

**Methods:**

Conceptualization and analysis of individual psychotherapy cases in the framework of psychodynamic approach.

**Results:**

Five typical patterns of anxiety, defensive functioning and mentalization structures were identified: Paranoial pattern is marked by flooding with persecutory anxiety, exaggerated subjective uncertainty. reflected in chaotic boundlessness, incoherence, fantasies of hostility, splitting and polarization of self and others. Depressive pattern reflects inability to sustain ambiguity due to deficiency of internal supports, overly dependence on others, conformity, obedience to authority and denial of personal standards and individuality. Noogenic pattern refers to negative affective states, generated by the ambiguity, contradictions and ambivalence of information. The means to regulate it include the lowering of the level of psychical functioning, with cognitive simplification, preference for order, routine and predictability. Transgression pattern suggests maniacal fascination with the dissipation of limits, normative restraints and rules, and the triumph of the narcissistic-perfectionist permissiveness. Constructivity pattern consists of the pleasure from explorations and insights, creation of new meanings, creative reappraisal and reconstruction of ambiguous situations.

**Conclusions:**

The typical patterns of experiencing ambiguity that were singled out may have diagnostic and prognostic significance in evaluating the individual resource potential in situations of COVID related dangers and isolation.

